# Haemoglobin status and predictors of anaemia among pregnant women in Mpigi, Uganda

**DOI:** 10.1186/1756-0500-7-712

**Published:** 2014-10-10

**Authors:** Sam Ononge, Oona Campbell, Florence Mirembe

**Affiliations:** Department of Obstetrics and Gynaecology, Makerere University College of Health Sciences, P.O. Box 7072, Kampala, Uganda; Department of Epidemiology and Population Health, London School of Hygiene and Tropical Medicine, Keppel Street, London, WC1E 7HT United Kingdom

**Keywords:** Haemoglobin, Anaemia, Pregnancy

## Abstract

**Background:**

Anaemia in pregnancy is a major public health problem especially in the low-income countries where it is highly prevalent. There has been no recent study in Uganda about the factors associated with anaemia in pregnancy. We aimed to assess the current haemoglobin (Hb) status and factors associated with anaemia (Hb < 11.0 g/dl) in pregnant women in Mpigi, Uganda.

**Methods:**

We assessed Hb levels of 2436 pregnant women at 28+ weeks of gestation at six health facilities, who were approached to participate in a stepped-wedge cluster-randomised trial of antenatal distribution of misoprostol (for self-administration after home birth or when oxytocin is not available). Women were administered a questionnaire and their baseline blood haemoglobin was examined using portable HemoCueR Hb 301 system. Predictors of anaemia were estimated using linear and logistic regression analysis.

**Results:**

The mean Hb was 11.5 (±1.38) g/dl and prevalence of anaemia (Hb < 11.0 g/dl) was 32.5% (95% CI 30.6%, 34.3%). After adjusting for measured confounders, factors associated with increased risk of anaemia in pregnancy were malaria infection (OR: 1.32, 95% CI: 1.11, 1.58), Human Immuno-deficiency Virus infection (OR: 2.13, 95% CI: 1.36, 2.90) and lack of iron supplementation (OR: 1.66, 95% CI: 1.36, 2.03). Intermittent presumptive treatment of malaria, maternal age and parity showed a weak association with anaemia in pregnancy

**Conclusion:**

The high prevalence of anaemia in pregnancy in our setting highlights the need to put more effort in the fight against malaria and HIV, and also ensure that pregnant women access iron supplements early in pregnancy.

## Background

Globally, anaemia in pregnancy is a public health problem [1]. It is estimated that more than half of the pregnant women in the world are anaemic, with resource-limited settings having a higher burden than high-income nations [1]. Anaemia in pregnancy increases the risk of maternal and perinatal morbidity and mortality, and is associated with premature labour and low birth weight [2–4]. Knowing the prevalence of anaemia in pregnancy is a useful measure of the health and nutritional status of pregnant women. At the health facility level, it is important for individual case management and planning of resources. Prior studies have documented the risk factors for anaemia in pregnancy to include malaria [5–7], Human Immunodeficiency virus (HIV) [7–10] and teenage pregnancies [11–13]. However, there is inconsistent evidence of an association between high parity (5+) and anaemia in pregnancy. While some studies show high parity increases risk [14, 15], others show no increased risk [16]. Effective interventions to prevent anaemia in pregnancy include preconception and prenatal iron supplementation, and intermittent presumptive treatment of malaria (IPT) [17].

According to the last two Uganda Demographic and Health Surveys, there was a decline in the prevalence of anaemia in pregnancy in Uganda from 41.2% to 30.5% between 2001 and 2011 respectively [18]. Beyond the high levels, we do not have recent information on the factors contributing to anaemia in pregnancy. This study aims to assess the haemoglobin levels and anaemia status of pregnant women attending antenatal clinic in their 3^rd^ trimester in rural district in Uganda and assess what factors influence this status.

## Methods

In the period 14^th^ February 2013 to 30th November 2013, the study approached pregnant women attending antenatal -care in six health centres in Mpigi district and requested them to participate in a community trial of self-administered misoprostol when delivery occurred at home or when oxytocin or misoprostol were not available during facility delivery. Mpigi district headquarters is 33 kilometres from Kampala, the capital city of Uganda. The majority of people in the district are of low socioeconomic status, with peasant farming and fishing as the main occupations. This paper is a cross-sectional description of prevalence of anaemia in pregnancy, and the baseline factors that influenced the haemoglobin status of study participants.

The study population were pregnant women at 28+ weeks of gestation, attending antenatal care, with no plans to leave the area during pregnancy or in the immediate postpartum. Women with a planned elective caesarean section were excluded. The recruitment of study participants was as follows: when pregnant women had assembled and had their routine antenatal health education, they were briefed about the study objectives and design. Eligible women were invited to participate, and if they agreed, to give written informed consent. Study participants were then interviewed face-to-face by trained research assistants. The pre-tested questionnaire was used to collect maternal age, education and occupation, marital status, religious affiliation, transport cost to the health facility and delivery plans. The study also inquired about parity, the gestation of the first antenatal visit, prophylactic medications received in the course of pregnancy (antihelminthics, IPT & iron supplementation), and episodes of malaria (self-reported fever or laboratory-tested) and anti-malarial treatment. The women’s gestational age was established using their last normal menstrual period (LNMP) or ultrasound scan estimation. In a few cases where we were unable to estimate gestational age by LNMP or ultrasound scan, fundal height estimation was used to approximate gestational age. Haemoglobin levels were measured at the same time using a portable HemoCueR Hb 301 system. The research assistants were trained to estimate haemoglobin using the HemoCue. Capillary blood was collected from participants using a finger prick method under sterile conditions. The first drop of blood was wiped away using alcohol sterile wipes, and the next drop was placed into the Hemocue curvette for immediate testing of haemoglobin.

Double data entry was done using EpiData 3.1 (EpiData Association, Odense, Denmark). The data were analysed using Stata version 12 (Stata Corp, College Station, TX, USA). Descriptive statistics were used to characterize the study participants and their haemoglobin level status. To adjust for the altitude of the study setting, 0.1 g/dl was subtracted from measured haemoglobin in accordance with WHO recommendations [19]. Anaemia was defined as a haemoglobin level in pregnancy of less than 11.0 g/dl [20].

Means of haemoglobin and proportions of women with anaemia across categories of each covariate were performed using univariate and multivariate linear or logistic regression models. Variables with *P* < 0.2 in the univariate analyses were retained in the multivariate logistic regression model to determine which factors were independently associated with haemoglobin status and anaemia in third trimester of pregnancy. Maternal age, parity, gestational age at enrolment, iron supplementation, IPT, malaria episodes in current pregnancy, and HIV sero-status were included in the multivariate model

Permission to carry out the study was granted by the School of Medicine Research and Ethics Committee at Makerere University, Kampala, Uganda, and the Uganda National Council for Science and Technology. The study was registered with the Pan African Clinical Trials Network (PACTR201303000459148).

## Results

A total of 2545 eligible women were approached, 2466 (96.9%) of whom consented to participate. Data for haemoglobin were obtained for 2436 women (98.8%). Table 1 characterizes the study participants and the mean haemoglobin levels of the women. The mean age was 24.5 (±5.6) years (range: 14–48 years), 88% (2141/2436) were married and average years of education were 8 (±3.0). Approximately one quarter (641/2436) of the participants were primigravida and mean parity was 3.1 (±2.1). The mean gestational age at the first prenatal visit was 22.2 (±6.5) weeks. The average gestation at enrolment in our study was 32.3 (±3.4) weeks. Approximately one-third (34.1%) of women had not started iron supplements before enrolling into the study. Women who had taken the recommended two doses of de-worming tablets and two doses of antimalarial prophylaxis were 31.4% and 38.2% respectively. One hundred and ninety (7.8%) of the participants were HIV positive and 9 (0.4%) had undetermined HIV sero-status. The women’s mean haemoglobin level at enrolment was 11.5 (±1.38) g/dl and median was 11.6 g/dl (range: 6.9 - 15.9 g/dl). Seven hundred eighty five (32.2%) had moderate anaemia (Hb 7.0-10.9 g/dl), and 0.3% had severe anaemia (Hb <7.0 g/dl).

**Table 1:** **Participants’ characteristics and the mean haemoglobin status of 2436 pregnant women**

Characteristic	Frequency	Percent (%)	Mean (sd) of Hb	95% CI	P- value*
Overall haemoglobin	2436	100	11.5 (1.38)	11.6-11.7	
Age in years					
Below 20	474	19.5	11.3 (1.33)	11.2-11.4	0.006
20-24	937	38.5	11.5 (1.40)	11.4-11.6	
25-29	680	27.9	11.6 (1.36)	11.5-11.7	
30-34	176	7.2	11.5 (1.52)	11.3-11.8	
≥35	169	6.9	11.5 (1.34)	11.3-11.7	
Marital status					
Single	295	12.1	11.4 (1.44)	11.2-11.5	0.055
Married	2141	87.9	11.5 (1.37)	11.5-11.6	
Education level					
No formal	77	3.2	11.8 (1.46)	11.5-12.1	0.437
Lower Primary (P1-4)	169	6.9	11.4 (1.38)	11.2-11.6	
Upper Primary (P5-7)	967	39.7	11.5 (1.37)	11.4-11.6	
Secondary (S1-S6)	1158	47.5	11.5 (1.40)	11.4-11.6	
Tertiary	65	2.7	11.5 (1.27)	11.1-11.8	
Parity					
Nulliparous	641	26.3	11.4 (1.37)	11.3-11.5	0.083
1	582	24.0	11.5 (1.40)	11.4-11.6	
2	414	17.0	11.6 (1.33)	11.5-11.7	
3	286	11.7	11.6 (1.39)	11.5-11.8	
4	228	9.4	11.5 (1.36)	11.5-11.6	
Para 5+	285	11.7	11.6 (1.43)	11.4-11.7	
Timing of 1^st^ visit					
First trimester	251	10.3	11.3 (1.35)	11.1-11.5	0.034
Second trimester	1505	61.8	11.5 (1.38)	11.5-11.6	
Third trimester	680	27.9	11.5 (1.40)	11.4-11.6	
Iron supplements (number of tablets)					
None	830	34.1	11.3 (1.38)	11.2-11.4	<0.001
1-30	1157	47.4	11.6 (1.37)	11.5-11.7	
31-60	302	12.5	11.6 (1.34)	11.5-11.9	
61+	147	6.0	11.8 (1.44)	11.5-11.9	
Doses of anti-helminthics taken					
Zero	511	21.0	11.4 (1.39)	11.3-11.6	0.15
One	1162	47.7	11.5 (1.37)	11.4-11.6	
Two	763	31.3	11.6 (1.38)	11.5-11.7	
Doses of antimalarial prophylaxis (IPT)					
Zero	433	17.8	11.4 (1.44)	11.3-11.6	0.004
One	1074	44.1	11.4 (1.36)	11.4-11.5	
Two	929	38.1	11.6 (1.37)	11.5-11.7	
Episodes of malaria					
None	1501	61.6	11.6 (1.35)	11.5-11.7	0.024
One	568	23.3	11.4 (1.44)	11.3-11.7	
Two	239	9.8	11.3 (1.40)	11.1-11.6	
Three	91	3.7	11.6 (1.36)	11.3-12.0	
Four +	37	1.5	11.3 (1.59)	10.9-11.9	
HIV sero-status					
Negative	2237	91.8	11.6 (1.36)	11.5-11.6	<0.001
Positive on ARVs	167	6.9	11.0 (1.52)	10.8-11.2	
Positive ARV naive	23	0.9	11.0 (1.38)	10.4-11.5	
Unknown	9	0.4	11.1 (1.28)	10.2-12.0	

Hb: Haemoglobin, IPT: Intermittent prophylactic treatment, ARVs: antiretroviral drugs. *p-values are from the F-test at univariate analysis.

In the univariate linear regression analysis (Table 1), there was a significant difference in haemoglobin level by various characteristics. Those less than 20 years and those above 34 years had a lower mean haemoglobin than those in the age range of between 20–34 years. Pregnant women who started attending ANC in their first trimester had a significantly lower mean haemoglobin level than the late starters. Women who had been on iron supplements had a higher mean haemoglobin than those that had not started taking any iron tablets. The pregnant women who had received two doses of anti-malarials for intermittent prophylaxis for malaria (IPT) had higher mean haemoglobin than those who had none or had taken only one dose. Women who had not had malaria had higher mean level than those who had suffered from malaria. Women who had received two doses of antihelminthics had slightly a higher mean haemoglobin than those who had taken one or zero antihelminthics. HIV positive women had lower mean haemoglobin than HIV negative women did.

In multivariate linear regression analysis (Table 2), gestational age at enrolment and iron supplementation were positively and independently associated with haemoglobin level while malaria and HIV infections were negatively associated with haemoglobin level.

**Table 2:** **Effects of different variables on haemoglobin status of pregnant women expressed as regression coefficients (β) with 95% CI**

		Crude analysis		Adjusted analysis
Characteristic	β (95% CI)	P-value	β (95% CI)	P- value*
Age in years				
Below 20	-0.21 (-0.36, -0.06)	0.006	-0.19 (-0.36, -0.02)	0.07
20-24	Ref			
25-29	0.11 (-0.03, 0.24)		0.10 (-0.06, 0.25)	
30-34	0.02 (-0.20, 0.24)		-0.03 (-0.28, 0.23)	
≥35	-0.03 (-.26, 0.19)		-0.05 (-0.34, 0.23)	
Parity				
Nulliparous	-0.20 (-0.37, -0.03)	0.08	-0.14 (-0.33, 0.06)	0.38
1	-0.12 (-0.30, 0.05)		-0.12 (-0.30, 0.05)	
2	Ref		Ref	
3	0.05 (-0.16, 0.26)		0.05 (-0.16, 0.26)	
4	-0.10 (-0.33, 0.12)		-0.12 (-0.35, 0.12)	
Para 5+	-0.01 (-0.22, 0.20)		0.03 (-0.22, 0.29)	
Timing of 1^st^ antenatal visit				
1^st^ trimester	Ref	0.03	Ref	0.05
2^nd^ trimester	0.24 (0.06, 0.43)		0.21 (0.03, 0.40)	
3^rd^ trimester	0.20 (0.00, 0.40)		0.26 (0.04, 0.48)	
Gestational age at enrolment	0.05 (0.03, 0.06)	<0.001	0.03 (0.01, 0.05)	<0.001
Iron supplements (number of tablets)				
None	Ref	<0.001		<0.001
1-30	0.27 (0.15, 0.39)		0.32 (0.19, 0.45)	
31-60	0.40 (0.22, 0.58)		0.37 (0.17, 0.58)	
61+	0.47 (0.23, 0.71)		0.53 (0.27, 0.80)	
Doses of antihelminthics taken				
Zero	-0.14 (-0.30, 0.01)	0.15	0.12 (-0.11, 0.35)	0.54
One	-0.10 (-0.22, 0.03)		0.09 (-0.10, 0.28)	
Two	Ref			
Doses of antimalarial prophylaxis (IPT)				
Zero	-0.21 (-0.37, -0.05)	0.004	0.03 (-0.20, 0.25)	0.20
One	-0.18 (-0.30, -0.06)		-0.12 (-0.30, 0.06)	
Two	Ref			
Episodes of malaria				
None	Ref	0.02		0.02
One	-0.13 (-0.26, 0.003)		-0.14 (-0.27, -0.01)	
Two	-0.29 (-0.48, -0.10)		-0.29 (-0.47, -0.10)	
Three	-0.01 (-0.30, 0.28)		0.01 (-0.29, 0.29)	
Four +	-0.19 (-0.64, 0.26)		-0.16 (-0.60, 0.29)	
HIV Sero-status				
Negative	Ref	<0.001		<0.001
Positive	-0.54 (-0.74, -0.33)		-0.63 (-0.85, -0.42)	

HIV: Human Immunodeficiency Virus; ARVs: Antiretroviral treatment.

*p-values are from the F-test after adjusting for variables that had a univariate p-value <0.2 (age, parity timing of first ANC visit, gestational age at enrolment, iron supplementation, antihelminthics, IPT malaria & HIV infection).

### Anaemia in third trimester of pregnancy

The study showed that in the third trimester, the prevalence of anaemia (Hb < 11.0 g/dl) was 32.5% (95% CI: 30.6%, 34.3%). The factors that had strong association with anaemia in pregnancy, as shown in Table 3, were malaria, HIV infection and iron supplementation. The effect of malaria episodes and HIV infection on anaemia increased after adjusting for potential confounders. Women who were on iron supplementation were less likely to have anaemia. Primigravida showed a weak positive association with anaemia but this was reduced in the adjusted model. Women who had fewer than the two doses of IPT showed a positive association with anaemia, but this was reduced in the adjusted model.

**Table 3:** **Factors associated with of anaemia in third trimester of pregnancy in Mpigi, Uganda**

			Crude analysis		Adjusted analysis*	
Characteristic	Frequency	Women with Hb < 11.0 g/dl n (%)	OR (95% CI) of Hb < 11.0 g/dl	P- value (trend)	OR (95% CI)	P value (trend)
Overall anaemia	2436	791 (32.5)				
Age in years						
Below 20	474	186 (39.2)	1.38 (1.10-1.74)	0.008 (0.016)	1.21 (0.93-1.58)	0.26(0.18)
20-24	937	298 (31.8)	1		1	
25-29	680	199 (29.3)	0.89 (0.72-1.10)		0.87 (0.68-1.12)	
30-34	176	52 (29.6)	0.90 (0.63-1.28)		0.86 (0.57-1.31)	
≥35	169	56 (31.1)	1.06 (0.75-1.51)		0.99 (0.63-1.57)	
Marital status						
Single	295	110 (37.3)	1.27 (0.98-1.64)	0.06	0.93 (0.70-1.21)	0.58
Married	2141	681 (31.8)	1		1	
Education level						
No formal	77	24 (31.2)	1	0.98 (0.94)		
Lower primary (P1-P4)	169	56 (33.1)	1.09 (0.61-1.95)			
Upper primary (P5-P7)	967	314 (32.5)	1.06 (0.64-1.75)			
Secondary(S1-S6)	1158	378 (32.6)	1.07 (0.65-1.76)			
Tertiary	65	19 (29.2)	0.91 (0.44-1.87)			
Parity						
Nulliparous	641	244 (38.1)	1.57 (1.21-2.06)	0.004 (0.024)	1.48 (1.08-2.04)	0.08 (0.13)
1	582	186 (32.0)	1.21 (0.92-1.59)		1.22 (0.91-1.63)	
2	414	116 (28.0)	1		1	
3	286	78 (27.3)	0.96 (0.69-1.35)		0.98 (0.69-1.39)	
4	228	79 (34.7)	1.36 (0.96-1.93)		1.42 (0.97-2.07)	
Para 5+	285	88 (30.1)	1.14 (0.82-1.60)		1.14 (0.76-1.71)	
Timing of 1^st^ visit						
First trimester	251	92 (36.7)	1	0.10 (0.9)	1	0.22 (0.15)
Second trimester	1504	466 (31.0)	0.78 (0.59-1.02)		0.79 (0.59-1.05)	
Third trimester	672	231 (34.4)	0.90 (0.67-1.22)		0.75 (0.54-1.05)	
Episodes of malaria						
Zero	1501	454 (30.3)	1	0.003	1	0.002
One or more	935	337 (36.0))	1.30 (1.09-1.54)		1.32 (1.11-1.58)	
HIV Sero-status						
Negative	2246	703 (31.3)	1	<0.001	1	<0.001
Positive	190	88 (44.0)	1.89 (1.40-2.55)		2.13 (1.56-2.90)	
Iron sulphate taken						
None	830	322 (38.8)	1.54 (1.29-1.83)	<0.001	1.66 (1.36-2.03)	<0.001
Yes	1606	469 (29.2)	1		1	
Doses of IPT						
Two	929	271 (29.2)	1	0.06	1	0.15
One or none	1507	520 (34.5)	1.28 (1.07-1.53)		1.15 (0.95-1.40)	
Doses of anti-helminthics taken						
One +	1925	617 (32.1)	1	0.39		
Zero	511	174 (34.1)	1.09 (0.89-1.35)			

Hb: Haemoglobin; OR: Odds ratios; IPT: Intermittent presumptive treatment of malaria.

P-values are from the likelihood ratio test, *adjusted for age, parity, marital status, iron tablets intake, IPT, malaria, timing of first antenatal visit and HIV-sero-status.

## Discussion

Among pregnant women in third trimester receiving antenatal clinic at Mpigi health facilities, the mean haemoglobin was 11.5 (±1.38) g/dl and the prevalence of anaemia (Hb < 11.0 g/dl) was 32.5% (95% CI 30.6-34.3). In the multivariable analysis, haemoglobin status of women was positively associated with early gestational age at enrolment or iron supplementation, and negatively associated with malaria and HIV infection. The odds of being anaemic in pregnancy were significantly associated with malaria (OR: 1.32, 95% CI: 1.11, 1.58), HIV (OR: 2.13, 95% CI: 1.56, 2.90) and lack of iron supplementation (OR: 1.66, 95% CI: 1.36, 2.03) after adjusting for measured confounders. A weak association found between parity and antimalarial prophylaxis (IPT) and anaemia disappeared after adjusting for confounders.

Not all pregnant women in Mpigi district attend antenatal clinics at the public health facilities, however, the Uganda Demographic Health Survey 2011 suggests that 94.9% of Ugandan women attend ANC at least once in pregnancy [21]. Moreover, from the district monthly reports, the number of pregnant women attending antenatal clinic at the government health facilities increased after free obstetric ultrasound scans were introduced for all the women, supported by STRIDES for family health, a USAID funded project. This attracts pregnant women and partners who are enthusiastic to know the status of the fetus in utero. Antenatal attendance is also encouraged by the TBAs surveyed prior to the trial, who stated they were less willing to attend to pregnant women who had not attended antenatal care at the health facility. Excluding the 79 pregnant women (planned for caesarean section birth, staying in island and moving out of Mpigi) is likely to have had a negligible effect on the overall prevalence of anaemia. For these reasons, and because of the high response rate, we feel our results are likely to be representative of the population of pregnant women in this area.

The methods for measuring Hb and the cut-offs for anaemia were as recommended by WHO [20]. Our assessment of malaria was based on a history of fever and of having received treatment of malaria from qualified medical personnel. While this may be better than studies that rely on women’s self-reports, we might still have overestimated the prevalence of malaria in pregnant women because there are many causes of fever in this country

The mean haemoglobin level of the pregnant women was higher than that reported by studies in Tanzania and Ghana [7, 22]. Both Tanzanian and Ghana studies enrolled women in the second trimester, a period in pregnancy when haemo-dilution occurs [23]. Our study recruited women in the third trimester when haemoglobin levels in normal women tend to rise slightly. Our study also showed that the later the gestational age at enrolment, the higher the haemoglobin level. Similarly higher haemoglobin levels were observed with iron supplementation. However, women who had malaria or HIV infections had a decrease in their haemoglobin level, as might be expected [8, 24, 25].

The prevalence of anaemia (Hb < 11.0 g/dl) in this population of pregnant women was high (32.5%) and was in the range of that reported in the Uganda Demographic and Health Survey (UDHS 2011 which was 30.6%) [21]. However, this was lower than the level of 39.7% obtained during an earlier 2003–2005 study in Uganda [8]. The difference could be due to the cut off for anaemia (Hb < 11.2, adjusted for altitude) used in the Muhangi study was higher than ours despite being at a similar altitude or because of improvements in socioeconomic status of the population over time. Socioeconomic status is a known determinant of anaemia [26, 27].

The study found an association between lack of iron supplementation and anaemia in pregnancy. Women who were not taking iron supplements had approximately a 60% higher odds of anaemia. This was different from a study in Cleveland, USA that did not find any effect of iron supplementation on reduction of anaemia in pregnancy [28]. The possible reason why the Cleveland study was not able to observe an association between iron supplementation and anaemia is that they enrolled only participants who had adequate iron stores or had no anaemia. The authors also noted in the paper a low power of the study due to loss-to-follow-up. This could have affected the effect estimate of iron supplementation on anaemia. However our finding are supported by a number of publications that document reduction in the level of anaemia in the ranges of 58%-83% when iron supplementation is given in randomized controlled trials [2, 29–31].

Our study also showed that malaria in pregnancy was strongly associated with anaemia. The relationship between malaria and anaemia in pregnancy has been documented in several studies [7, 8, 32]. These findings call for more effort to be put into malaria preventive strategies in the community in order for us to reduce the effects of morbidity and mortality related to anaemia.

Results in our study showed that pregnant women with HIV infection were twice more likely to have anaemia than HIV negative. A number of studies have documented HIV as a cause of anaemia in pregnancy [7, 9, 24, 25, 33–35]. HIV infection is associated with lower levels of serum folate and serum ferritin [10]. It is also suggested that HIV infection causes anaemia through changes in cytokine production, altered erythropoietin response to bone marrow and use of antiretroviral drugs, especially Zidovudine [24]

## Conclusion

Our study showed a decreasing prevalence of anaemia with increasing iron supplements used by the pregnant woman. This demonstrated that even in real life setting, iron supplementations in pregnant women appears to reduce the risk of anaemia. Nevertheless, 34.1% of women in our study had taken no iron supplementation, and coverage of IPT was also low (29.2%). We need to undertake additional measures to enable more pregnant women to start iron supplementation from the first trimester. This is possible by creating awareness through the village health teams (VHT) who can also be a channel for iron distribution.

Malaria and HIV in pregnancy were significantly associated with anaemia pregnancy. There is a need to reinforce malaria and HIV prevention strategies in the country, for example sleeping under insecticide treated nets for and behavioural change. There is a need to reactivate the 1990’s vigour in prevention of HIV/AIDs in the communities, where Government openly acknowledged HIV/AIDS as a public health problem and supported public media campaign that ensued to mobilise communities to the fight against HIV/AIDS.

## Abbreviations

AIDS: Acquired immuno-deficiency syndrome
ANC: Antenatal care
ART: Antiretroviral therapy
Hb: Haemoglobin
HIV: Human immune-deficiency virus
IPT: Intermittent presumptive treatment
LNMP: Last normal menstrual period
TBA: Traditional birth attendants
USA: United States of America
VHT: Village health teams
WHO: World Health Organisation.
